# A transcriptomic dataset comparing two methods of hepatocyte differentiation from human induced pluripotent stem cells

**DOI:** 10.1016/j.dib.2022.108477

**Published:** 2022-07-18

**Authors:** Xiugong Gao, Rong Li, Jeffrey J. Yourick, Robert L. Sprando

**Affiliations:** Division of Toxicology, Office of Applied Research and Safety Assessment, Center for Food Safety and Applied Nutrition, U.S. Food and Drug Administration, Laurel, MD 20708, USA

**Keywords:** Induced pluripotent stem cells, Hepatocyte differentiation, Hepatocyte-like cells, Transcriptomics, Microarray

## Abstract

A variety of methods have been reported for the differentiation of hepatocyte-like cells (HLCs) from human induced pluripotent stem cells (iPSCs) using various growth factors or small molecules. However, direct comparison of the differentiation efficiency and the quality of the final HLCs between different methods has rarely been reported. To fill this data gap, we compared two hepatocyte differentiation methods, termed Method 1 and Method 2, and published the major findings in a research article entitled “Phenotypical, functional and transcriptomic comparison of two modified methods of hepatocyte differentiation from human induced pluripotent stem cells” (Li et al., 2022). The current data article describes the transcriptomic dataset comparing the two methods. HLCs were collected at early maturation (day 17) and late maturation (day 21) stages of the differentiation and total RNA were isolated. Global gene expression profiling of the HLCs was conducted using Affymetrix GeneChip PrimeView Human Gene Expression Arrays. Primary human hepatocytes (PHHs) were also included for comparison. The microarray dataset has been deposited in the Gene Expression Omnibus of the National Center for Biotechnology Information with accession number GSE187011. Detailed interpretation and discussion of the data can be found in the corresponding research article (Li et al., 2022). This dataset is useful in providing a molecular basis for the differences observed between the two differentiation methods, offering new insights into gene regulations in hepatogenesis *in vitro*, and suggesting ways to further improve hepatocyte differentiation in order to obtain more mature HLCs for biomedical applications.

## Specifications Table

**Table:** 

Subject	Biological sciences/Omics: Transcriptomics
Specific subject area	Hepatocyte differentiation from human iPSCs
Type of data	Microarray gene expression data
How the data were acquired	Data were acquired on Affymetrix GeneChip PrimeView Human Gene Expression Arrays*.*
Data format	Processed microarray data in CEL and CHP formats
Description of data collection	Hepatocytes were differentiated from human iPSCs using two modified methods, Method 1 and Method 2*.* The resultant HLCs were collected at day 17 and day 21 of the differentiation and total RNA were isolated. PHHs were also included for comparison. Global gene expression profiling of the samples was conducted using Affymetrix GeneChip PrimeView Human Gene Expression Arrays. The array chips were scanned on Affymetrix GeneChip Scanner 3000 7G and the raw image DAT files were postprocessed using the Affymetrix GeneChip Command Console software v.4.0 to generate cell intensity CEL files. The CEL files were further processed using the Affymetrix Expression Console software v.1.3 to generate probe intensity CHP files.
Data source location	U.S. Food and Drug Administration Laurel, MD USA
Data accessibility	The dataset is publicly available at the Gene Expression Omnibus of the National Center for Biotechnology Information with accession number GSE187011. https://www.ncbi.nlm.nih.gov/geo/query/acc.cgi?acc=GSE187011
Related research article	R. Li, Y. Zhao, J.J. Yourick, R.L. Sprando, X. Gao, Phenotypical, functional and transcriptomic comparison of two modified methods of hepatocyte differentiation from human induced pluripotent stem cells, Biomed Rep (2022) 16(5):43. doi: 10.3892/br.2022.1526.

## Value of the Data

• The data reflect direct transcriptomic comparison of different methods of hepatocyte differentiation from human iPSCs [1].
• The data are interesting to the general biomedical research community and are especially beneficial to researchers in the fields of developmental biology and regenerative medicine.
• The data may offer new insights into gene regulations in hepatogenesis, and suggest ways to further improve hepatocyte differentiation in order to obtain more mature HLCs for biomedical applications.

## 1 Data Description

The dataset consists of 4 sample types collected from two hepatocyte differentiation methods, Method 1 and Method 2, at two time points, day 17 and day 21. An additional sample type, PHHs, was also included for comparison. Each sample type has 3 biological replicates. Therefore, totally 15 samples were contained in the dataset (Table 1). For sample names in the table and in the figures that follow, the following abbreviations are used: M1, Method 1; M2, Method 2; D17, day 17; D21, day 21.

**Table 1:** List of samples in the dataset.

Sample name	title	CEL file	CHP file
M1_D17_1	HLCs generated by M1 at D17, biological rep1	M1_D17_1.CEL	M1_D17_1.CHP
M1_D17_2	HLCs generated by M1 at D17, biological rep2	M1_D17_2.CEL	M1_D17_2.CHP
M1_D17_3	HLCs generated by M1 at D17, biological rep3	M1_D17_3.CEL	M1_D17_3.CHP
M1_D21_1	HLCs generated by M1 at D21, biological rep1	M1_D21_1.CEL	M1_D21_1.CHP
M1_D21_2	HLCs generated by M1 at D21, biological rep2	M1_D21_2.CEL	M1_D21_2.CHP
M1_D21_3	HLCs generated by M1 at D21, biological rep3	M1_D21_3.CEL	M1_D21_3.CHP
M2_D17_1	HLCs generated by M2 at D17, biological rep1	M2_D17_1.CEL	M2_D17_1.CHP
M2_D17_2	HLCs generated by M2 at D17, biological rep2	M2_D17_2.CEL	M2_D17_2.CHP
M2_D17_3	HLCs generated by M2 at D17, biological rep3	M2_D17_3.CEL	M2_D17_3.CHP
M2_D21_1	HLCs generated by M2 at D21, biological rep1	M2_D21_1.CEL	M2_D21_1.CHP
M2_D21_2	HLCs generated by M2 at D21, biological rep2	M2_D21_2.CEL	M2_D21_2.CHP
M2_D21_3	HLCs generated by M2 at D21, biological rep3	M2_D21_3.CEL	M2_D21_3.CHP
PHH_1	PHHs, biological rep1	PHH_1.CEL	PHH_1.CHP
PHH_2	PHHs, biological rep2	PHH_2.CEL	PHH_2.CHP
PHH_3	PHHs, biological rep3	PHH_3.CEL	PHH_3.CHP

The raw image DAT files generated by the scanner, which contain pixel intensity values, were postprocessed using the Affymetrix GeneChip Command Console software v.4.0 to generate cell intensity CEL files, which contain information of probe-level intensity values. The values of individual probes belonging to each probe set in the CEL files were then summarized using the robust multi-array average (RMA) algorithm [2] embedded in the Affymetrix Expression Console software v.1.3, which comprises of convolution background correction, quantile normalization, and median polish summarization, to generate CHP files. CHP files contain information of probe set or gene-level intensity values.

**Fig. 1:**
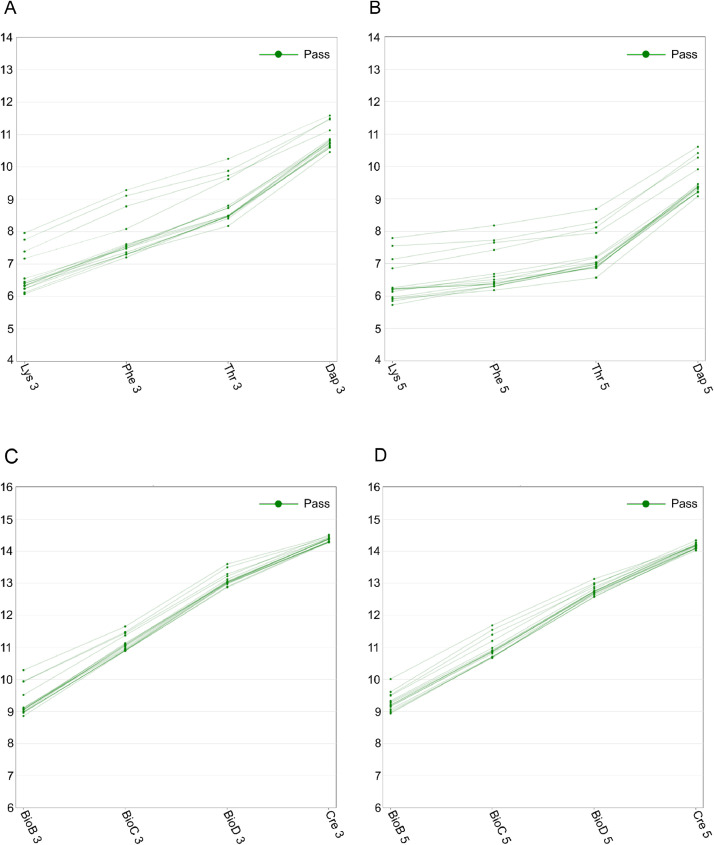
Metrics of labeling and hybridization controls indicating all samples were within boundaries. (A,B) Labeling controls at 3’ and 5’ ends, respectively. (C,D) Hybridization controls at 3’ and 5’ ends, respectively. Each dot represents a sample. For all the samples, the correct rank order of the signal values is shown for the labeling probe sets (Lys < Phe < Thr < Dap) and for the hybridization probe sets (BioB < BioC < BioD < Cre). Green color indicates a sample passed the quality control criteria.

For each sample, both the CEL and the CHP files are included in the dataset. All the samples were assessed for data quality using the Affymetrix Expression Console software v.1.3 and all quality control metrics (including spike-in controls during target preparation and hybridization and housekeeping gene controls) were found within boundaries. Metrics of labeling and hybridization controls are shown in Fig. 1.

**Fig. 2:**
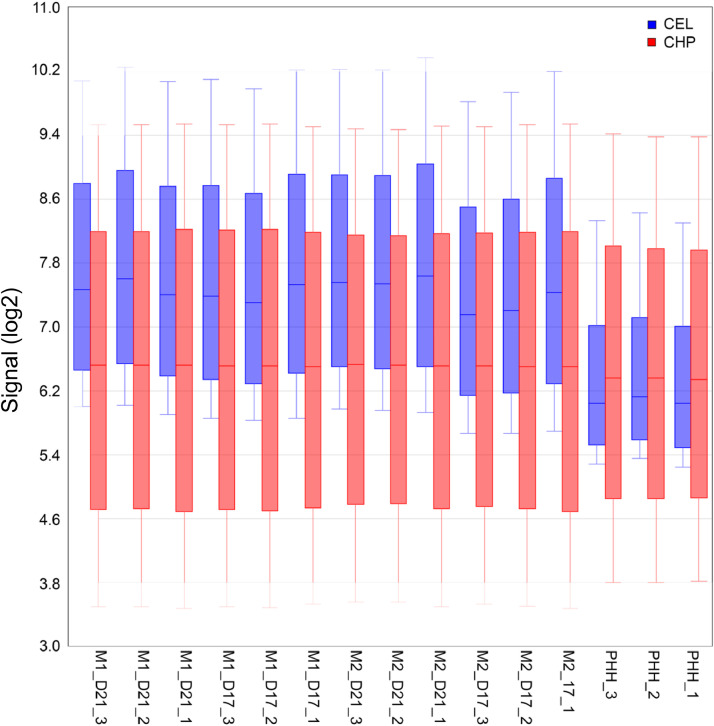
Boxplot showing signal intensity (log2 scale) of the samples before and after summarization. A batch effect was observed at the cell intensity CEL level but was largely removed at the probe set CHP level, after RMA normalization and summarization.

The data for the HLCs and for the PHHs were collected at different times, therefore a batch effect was observed at the cell intensity (CEL) level. However, the batch effect was largely eliminated at the probe set (CHP) level after normalization and summarization using RMA (Fig. 2).

**Fig. 3:**
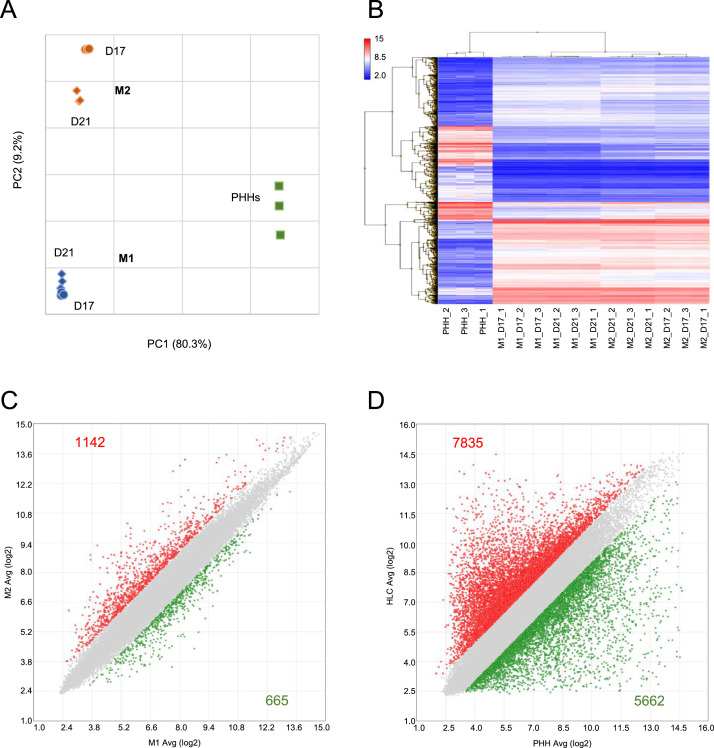
Unsupervised exploratory data analysis. (A,B) PCA and HCA, respectively, show that there is clear separation between the different cell types (HLCs vs. PHHs), the different differentiation methods (M2 vs. M1), and the different time points (D21 vs. D17). (C,D) Scatter plots showing the DEGs, using |fold change| > 2 and *p*-value < 0.05, between the two differentiation methods (M2 vs. M1) and between the two cell types (HLCs vs. PHHs), respectively. The numbers of upregulated genes are shown in red and downregulated genes in green.

Results of unsupervised exploratory data analysis are shown in Fig. 3. There is clear separation between the different cell types, the different differentiation methods, and to a lesser extent between the different time points, as shown in the principal component analysis (PCA) plot (Fig. 3A) and the hierarchical clustering analysis (HCA) dendrogram (Fig. 3B). A total of 1807 differentially expressed genes (DEGs) were identified between the two differentiation methods (Fig. 3C); in comparison, 13,497 DEGs were found between the two cell types (Fig. 3D). Details of supervised data analysis could be found in the related research article [1].

## 2 Experimental Design, Materials and Methods

### 2.1 Experimental Design

HLCs were differentiated from a human iPSC line using two methods, Method 1 and Method 2. The resultant HLCs were collected at early maturation (day 17) and late maturation (day 21) stages of the differentiation. Cryopreserved PHHs were included in the study for comparison with the HLCs. Total RNA was isolated from the cells and global gene expression profiling of the samples was conducted using Affymetrix GeneChip PrimeView Human Gene Expression Arrays.

### 2.2 Human iPSC Line and Single-Cell Culture of iPSCs

Human iPSC cell OARSAi002-A was previously generated in our laboratory from cord blood of a healthy non-Hispanic white male using self-replicative RNA reprogramming technology [3]. Cells were maintained as single-cell culture (no colony formation) on COAT-1 pre-coated 6-well tissue culture plate in Cellartis DEF-CS Culture System (Takara Bio USA, Mountain View, CA) at 37 °C, 5% CO_2_ incubator. Details of cell culture and passage were reported previously [1].

### 2.3 Hepatocytes Differentiation from iPSCs

iPSCs were first induced to definitive endoderm using the STEMdiff Definitive Endoderm Kit from STEMCELL Technologies (Vancouver, Canada) following the manufacturer's instructions. Hepatic specification and maturation were then carried out using the two methods, Method 1 and Method 2, modified from previously published methods. Details of the differentiation methods were reported previously [1].

### 2.4 RNA Extraction and Quality Assurance

Cells were harvested on day 17 and day 21 of hepatocyte differentiation and stored at −80 °C before RNA extraction. Cells were lysed in RLT buffer (Qiagen, Valencia, CA) and homogenized using QIAshredder (Qiagen). Total RNA was extracted from the cell lysates using EZ1 RNA Cell Mini Kit (Qiagen) on EZ1 Advanced XL automated RNA purification instrument (Qiagen) following the manufacturer's instruction. An on-column DNase digestion step was included to remove potentially available contaminating DNA. Total RNA concentration and purity (260/280) were subsequently measured using a NanoDrop 2000 UV-Vis spectrophotometer (NanoDrop Products, Wilmington, DE). The integrity of RNA samples was further assessed using the Agilent 2100 Bioanalyzer with the RNA 6000 Nano Reagent Kit (Agilent Technologies) to obtain the RNA integrity number.

### 2.5 RNA Processing and Microarray Experiment

All reagents and instruments used in the microarray experiment were obtained from Affymetrix (Santa Clara, CA). Total RNA samples were processed using GeneChip 3′ IVT PLUS Reagent Kit and hybridized onto GeneChip PrimeView Human Gene Expression Arrays following protocols from the manufacturer. Briefly, single-stranded complementary DNA (cDNA) was generated from 100 ng total RNA using reverse transcriptase and a T7-linked oligo(dT) primer, which was then converted to double-stranded cDNA using DNA polymerase and RNase H. Subsequently, complementary RNA (cRNA) was synthesized through *in vitro* transcription (IVT) with biotinylated UTP and CTP, using T7 RNA polymerase as the enzyme and the second strand of the double-stranded cDNA as the template.

The biotin-labeled cRNA was then purified and a fraction of 12 µg was fragmented by Mg^2+^ at 94 °C. Fragmented cRNA was then hybridized onto the microarray chips in the GeneChip Hybridization Oven 645 at 45 °C for 16 h. After hybridization, the microarray chips were stained and washed on the GeneChip Fluidics Station 450. Finally, the chips were scanned using GeneChip Scanner 3000 7G, and the scanned image (DAT) files were further preprocessed using Affymetrix GeneChip Command Console software (v. 4.0) to produce cell intensity (CEL) files. Arrays were quality-checked using the Affymetrix Expression Console software (v. 1.3) prior to data processing and analysis in the next step.

### 2.6 Microarray Data Processing and Unsupervised Data Analysis

The robust multi-array average (RMA) algorithm integrated in the Affymetrix Expression Console software (v.1.3) were used to summarize values of individual probes belonging to one probe set in the CEL files to generate the CHP files. Unsupervised principal component analysis (PCA) and hierarchical cluster analysis (HCA) on normalized data from all samples were then performed using the ArrayTrack software system developed by the U.S. FDA [4] to explore similarities and differences among the samples. Subsequently, one-way analysis of variance (ANOVA) was conducted using the Affymetrix Transcriptome Analysis Console (TAC) software (v.4.0) to identify differentially expressed genes (DEGs). For each comparison between two experimental groups, the selection of DEGs were based on the fold change (FC) of each annotated gene coupled with its corresponding *p*-value using the cutoff values of |FC| > 2.0 and *p* < 0.05.

## Ethics Statements

This manuscript adheres to the Elsevier Ethics in publishing standards.

## CRediT authorship contribution statement

**Xiugong Gao:** Conceptualization, Methodology, Investigation, Formal analysis, Writing – original draft, Visualization, Supervision, Project administration. **Rong Li:** Conceptualization, Methodology, Investigation. **Jeffrey J. Yourick:** Resources, Funding acquisition, Writing – review & editing. **Robert L. Sprando:** Resources, Funding acquisition, Writing – review & editing.

## Declaration of Competing Interest

The authors declare that they have no known competing financial interests or personal relationships that could have appeared to influence the work reported in this paper.

## Data Availability

Expression data from hepatocyte-like cells (HLCs) differentiated from human induced pluripotent stem cells (iPSCs) (Original data) (Gene Expression Omnibus).
